# Supplementary material for: Non-cell-autonomous activation of IL-6/STAT3 signaling mediates FGF19-driven hepatocarcinogenesis
Source: Nat Commun. 2017 May 16;8:15433. doi: 10.1038/ncomms15433 (PMC5440856; doi:10.1038/ncomms15433)
Supplement: Supplementary Information — Supplementary Figures and Supplementary Tables [file ncomms15433-s1.pdf]

a

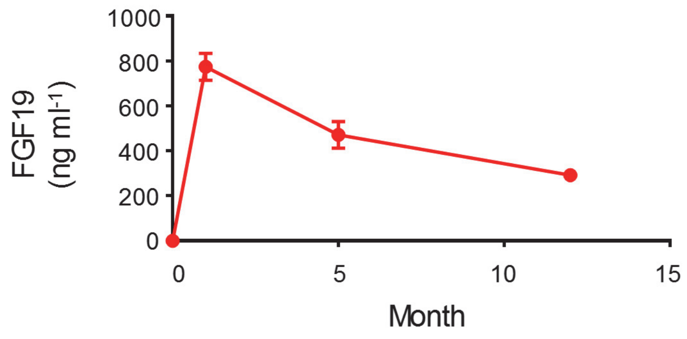
FGF19
(ng ml-1)
1000
800
600
400
200
0
0
5
10
15
Month

b

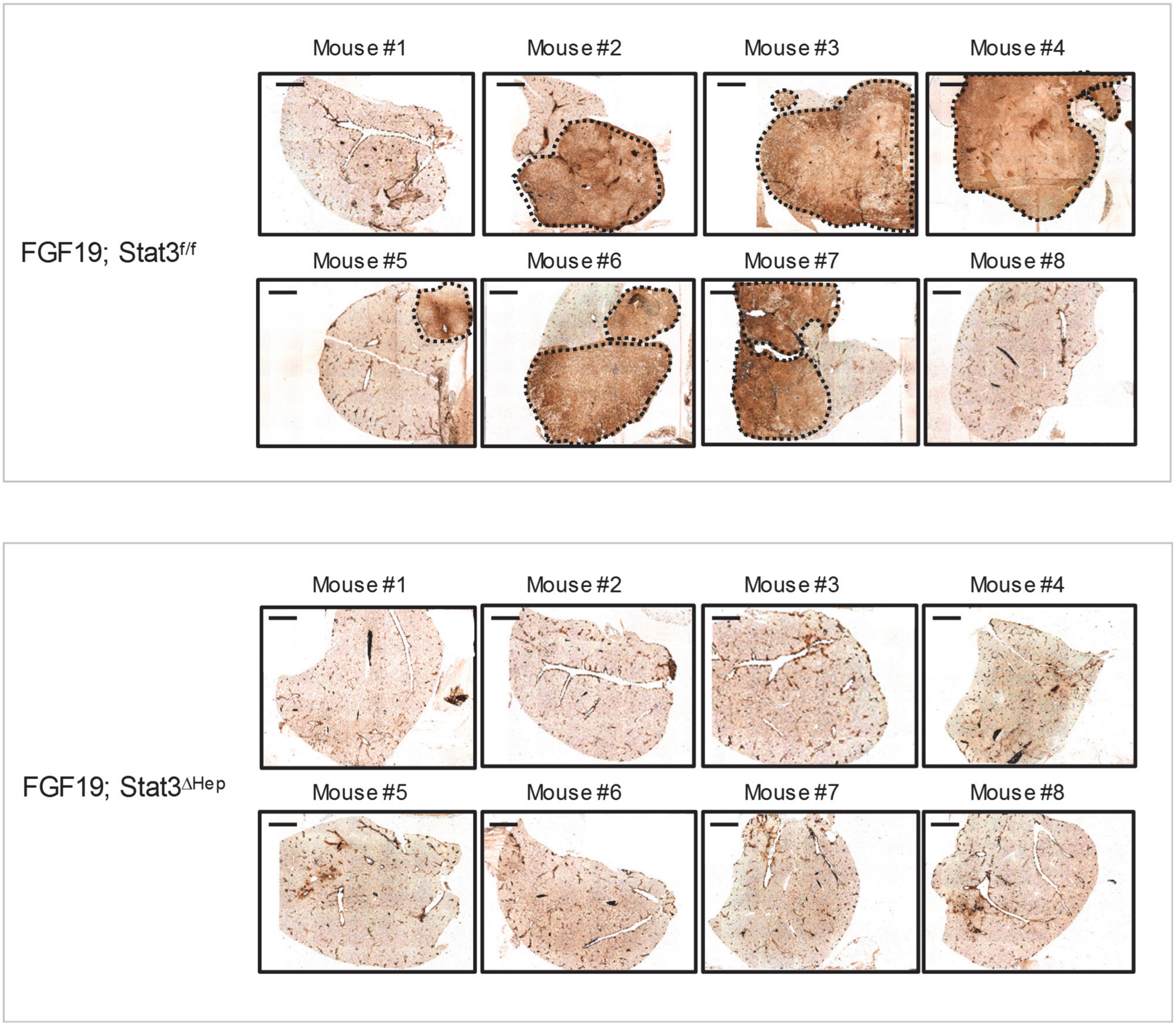
FGF19; Stat3f/f
Mouse #1
Mouse #2
Mouse #3
Mouse #4
Mouse #5
Mouse #6
Mouse #7
Mouse #8
FGF19; Stat3ΔHep
Mouse #1
Mouse #2
Mouse #3
Mouse #4
Mouse #5
Mouse #6
Mouse #7
Mouse #8

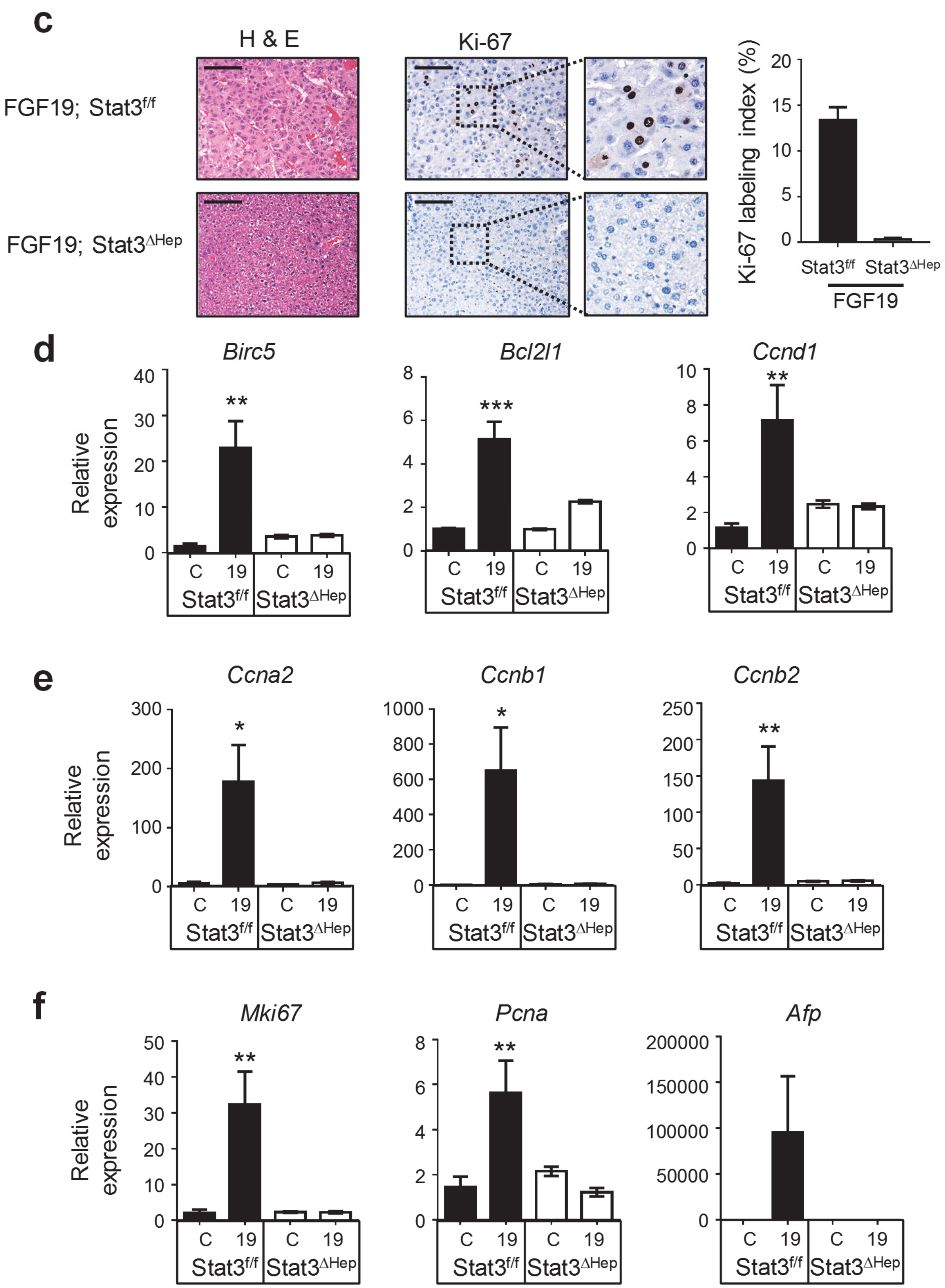
c
H & E
Ki-67
FGF19; Stat3f/f
FGF19; Stat3ΔHep
Ki-67 labeling index (%)
20
15
10
5
0
Stat3f/f
Stat3ΔHep
FGF19
d
Birc5
Bcl2l1
Ccnd1
Relative expression
40
30
20
10
0
**
8
6
4
2
0
***
10
8
6
4
2
0
**
C
19
C
19
Stat3f/f
Stat3ΔHep
e
Ccna2
Ccnb1
Ccnb2
Relative expression
300
200
100
0
*
1000
800
600
400
200
0
*
250
200
150
100
50
0
**
C
19
C
19
Stat3f/f
Stat3ΔHep
f
Mki67
Pcna
Afp
Relative expression
50
40
30
20
10
0
**
8
6
4
2
0
**
200000
150000
100000
50000
0
C
19
C
19
Stat3f/f
Stat3ΔHep

Supplementary Figure 1. Hepatocyte-specific ablation of STAT3 eliminates FGF19-induced pro-tumorigenic gene expression. (**a**) Time course of circulating FGF19 levels in *Stat3*$^{f/f}$ mice following AAV-FGF19 administration. 14 to 18 week-old *Stat3*$^{f/f}$ mice (n = 8) received a single tail vein injection of AAV-FGF19. Blood samples were taken prior to AAV injection, and at 1, 5, and 12 months after AAV injection. FGF19 levels were determined by ELISA. (**b**) Tumor area for individual mouse by immunohistochemistry. Liver sections were stained with anti-glutamine synthetase and DAB substrates (brown color). Tumors are indicated by dashed lines; scale bars, 5 mm. (**c**) Immunohistochemical staining with antibodies against Ki67, a marker of proliferation. Note that FGF19 increases Ki-67-positive cells in *Stat3*$^{f/f}$ mice, but not *Stat3*$^{\Delta Hep}$ mice. Scale bars, 200 µm. n = 8 mice per group. Quantification of Ki-67 labeling index is shown as a bar graph. (**d**) STAT3 target genes, *Birc5 (Survivin), Bcl2l1 (Bcl-x$_L$*), and *Ccnd1* (*Cyclin D1)*, were induced by FGF19 (19) in *Stat3*$^{f/f}$ mice, but not *Stat3*$^{\Delta Hep}$ mice. Mice were euthanized 12 months after AAV-FGF19 administration. qRT-PCR analysis were performed on RNA samples extracted from the liver. C, control virus. (**e**) Cyclins were induced by FGF19 in *Stat3*$^{f/f}$ mice, but not *Stat3*$^{\Delta Hep}$ mice. (**f**) FGF19 induces proliferative and HCC-related genes in *Stat3*$^{f/f}$ mice, but not *Stat3*$^{\Delta Hep}$ mice. Values are mean $\pm$ s.e.m. ***P < 0.001, **P < 0.01, *P < 0.05 versus control virus-injected *Stat3*$^{f/f}$ mice by one-way ANOVA.

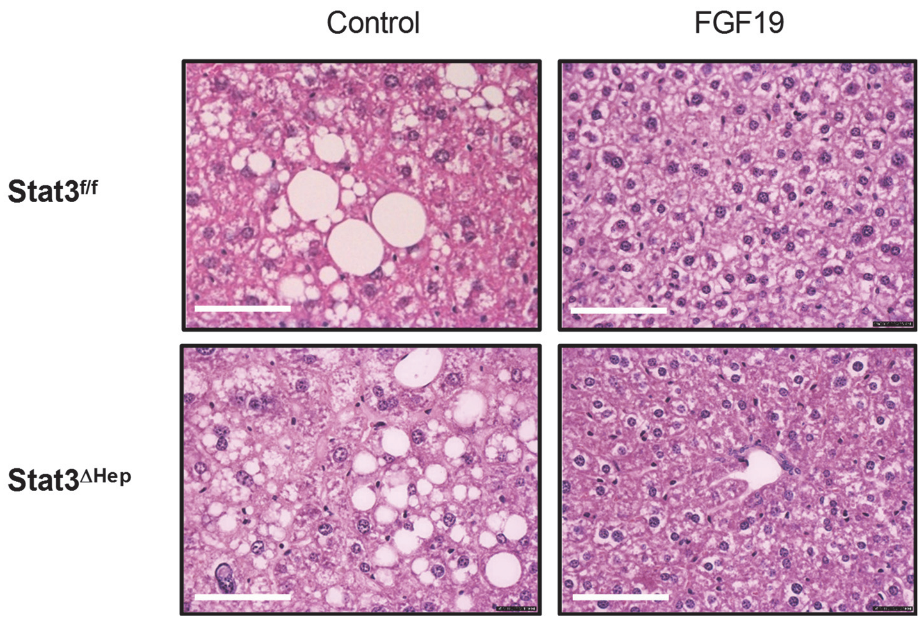

Supplementary Figure 2. Loss of STAT3 in hepatocytes does not impair FGF19-dependent improvement in steatosis. *Stat3*$^{f/f}$ or *Stat3*$^{\Delta Hep}$ mice were euthanized 12 months after a tail vein injection of AAV-FGF19 (n = 8) or a control virus (n = 5). Representative H & E images of liver sections are shown. Note vacuoles (steatosis) present in both *Stat3*$^{f/f}$ and *Stat3*$^{\Delta Hep}$ mice treated with the control virus, but not in FGF19-treated mice. Scale bars, 100 μm.

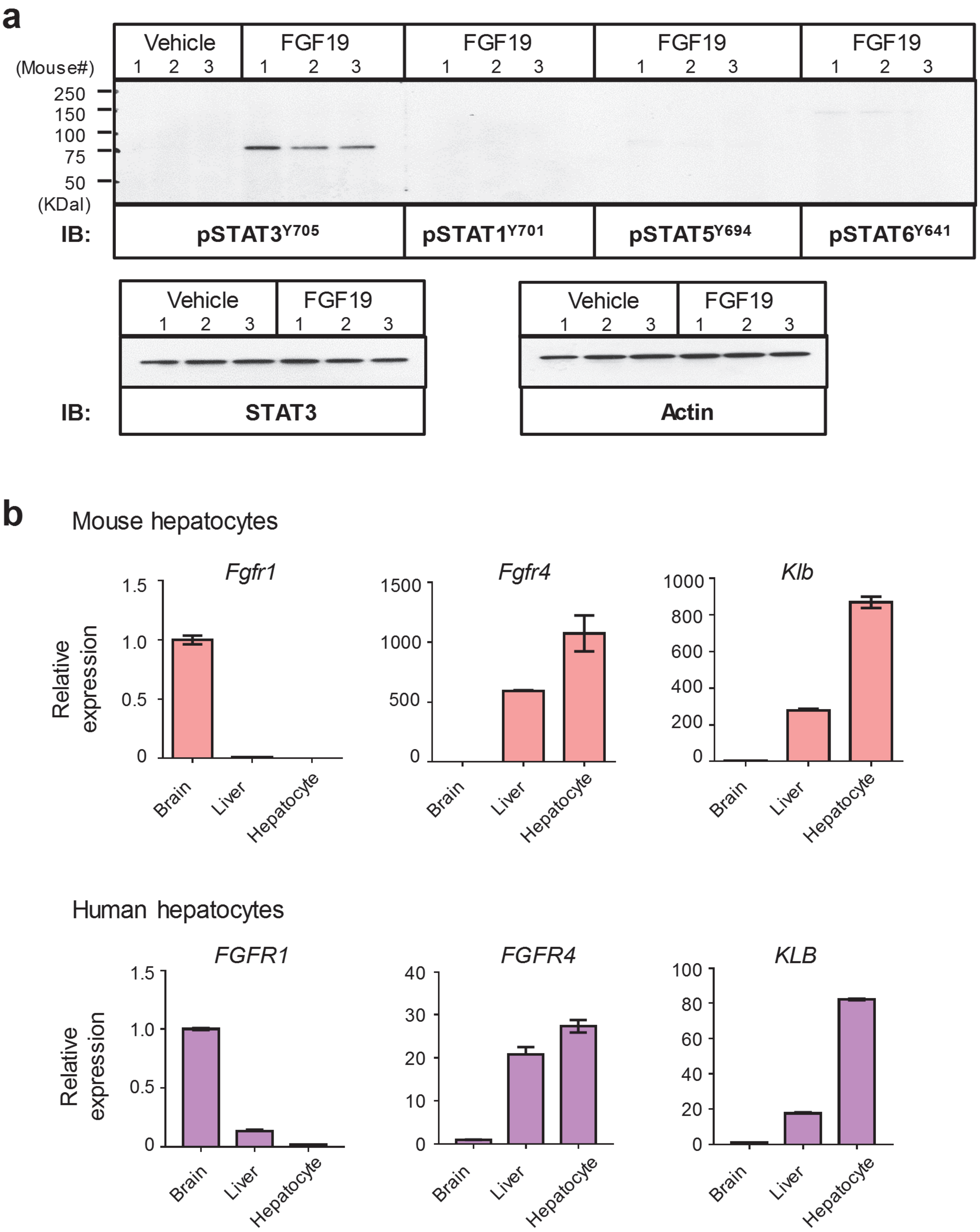
a
Vehicle
FGF19
FGF19
FGF19
FGF19
(Mouse#)
1 2 3
250
150
100
75
50
(KDal)
IB:
pSTAT3Y705
pSTAT1Y701
pSTAT5Y694
pSTAT6Y641
Vehicle
FGF19
STAT3
Actin
b
Mouse hepatocytes
Fgfr1
Fgfr4
Klb
Relative expression
Brain
Liver
Hepatocyte
1.5
1.0
0.5
0
1500
1000
500
800
600
400
200
Human hepatocytes
FGFR1
FGFR4
KLB
40
30
20
10
100
80
60

Supplementary Figure 3. Specificity of FGF19-induced STAT3 phosphorylation in vivo and receptor expression in isolated hepatocytes. (**a**) Phosphorylation of STAT3 at tyrosine residue 705 (pSTAT3^{Y705}), but not the conserved tyrosine residue in other STAT isoforms, was induced by FGF19. 11~12-week old *db/db* mice (*n* = 3 per group) received a single intraperitoneal injection of 1 mg/kg FGF19 or vehicle, and livers were harvested 2 hours post dose. Shown are immunoblot analyses of liver lysates with antibodies against pSTAT3^{Y705}, pSTAT1^{Y701}, pSTAT5^{Y694}, pSTAT6^{Y641}, STAT3 and anti-β-actin. (**b**) Primary hepatocytes were isolated by collagenase digestion method from mouse or human livers. Real-time quantitative RT-PCR was performed to determine mRNA levels of FGFR1, FGFR4, and KLB receptors. Data are normalized to housekeeping gene GAPDH, and are relative to the expression in the brain.

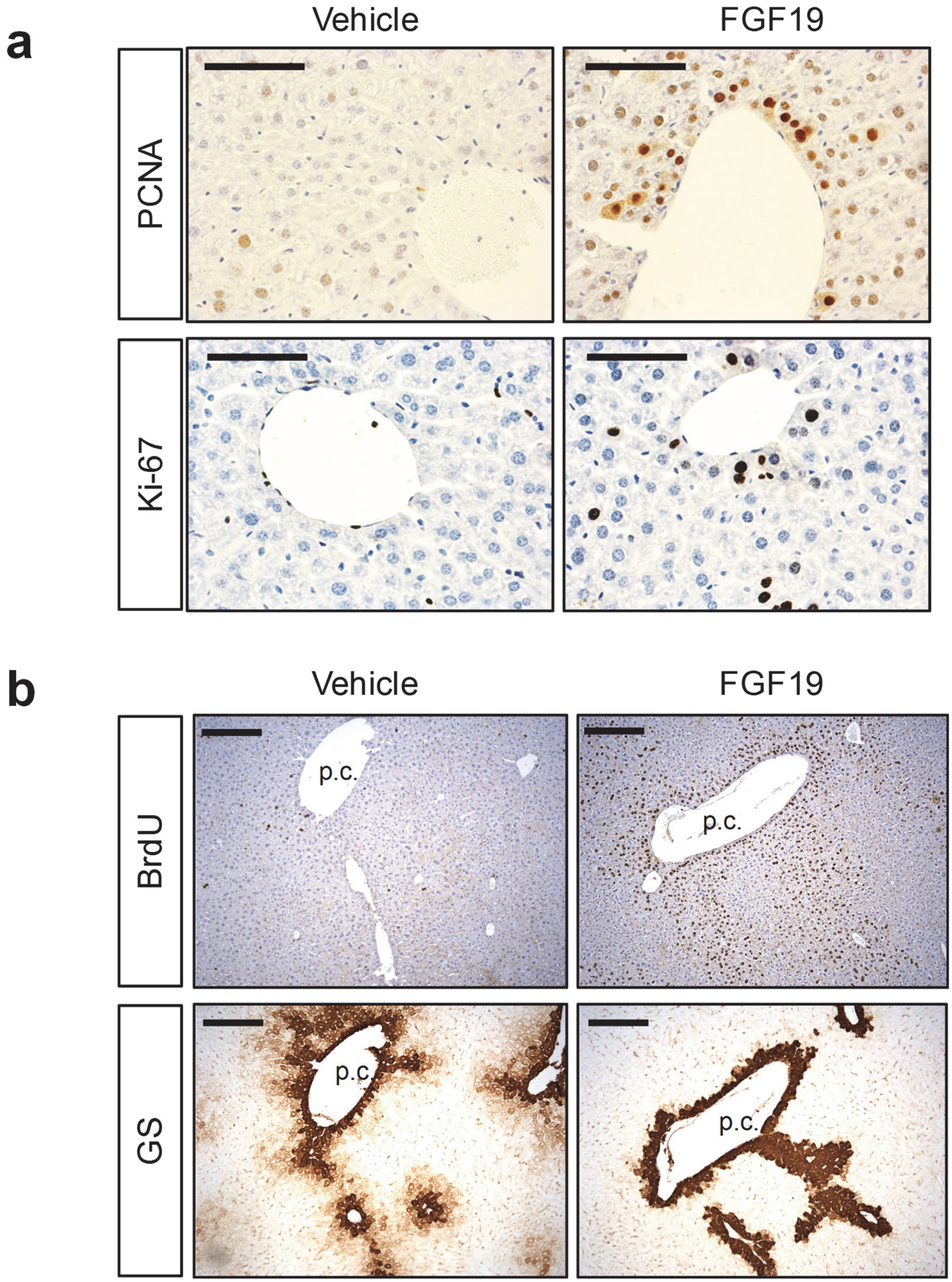
a
Vehicle
FGF19
PCNA
Ki-67
b
Vehicle
FGF19
BrdU
p.c.
p.c.
GS
p.c.
p.c.

Supplementary Figure 4. Non-cell-autonomous promotion of hepatocellular proliferation by FGF19. (**a**) FGF19 increases PCNA and Ki-67 staining in the liver. Mice (*n* = 5 per group) were implanted with osmotic pumps releasing BrdU (8.5 mg/kg/day) and FGF19 protein (0.4 mg/kg/day). Livers were harvested 6 days post implant. Shown are representative images of livers stained with anti-PCNA or anti-Ki-67 antibodies followed by DAB substrates (brown color). Sections were counter-stained with hematoxylin. Scale bars, 100 μm. (**b**) FGF19 increases proliferation of peri-central hepatocytes. Adjacent liver sections from mice implanted with osmotic pumps releasing BrdU and FGF19 were stained with anti-BrdU (top panels) or anti-glutamine synthetase (marker of peri-central hepatocytes, bottom panels). DAB substrates (brown color) were used for color development. *p.c.*, peri-central. Scale bars, 100 μm.

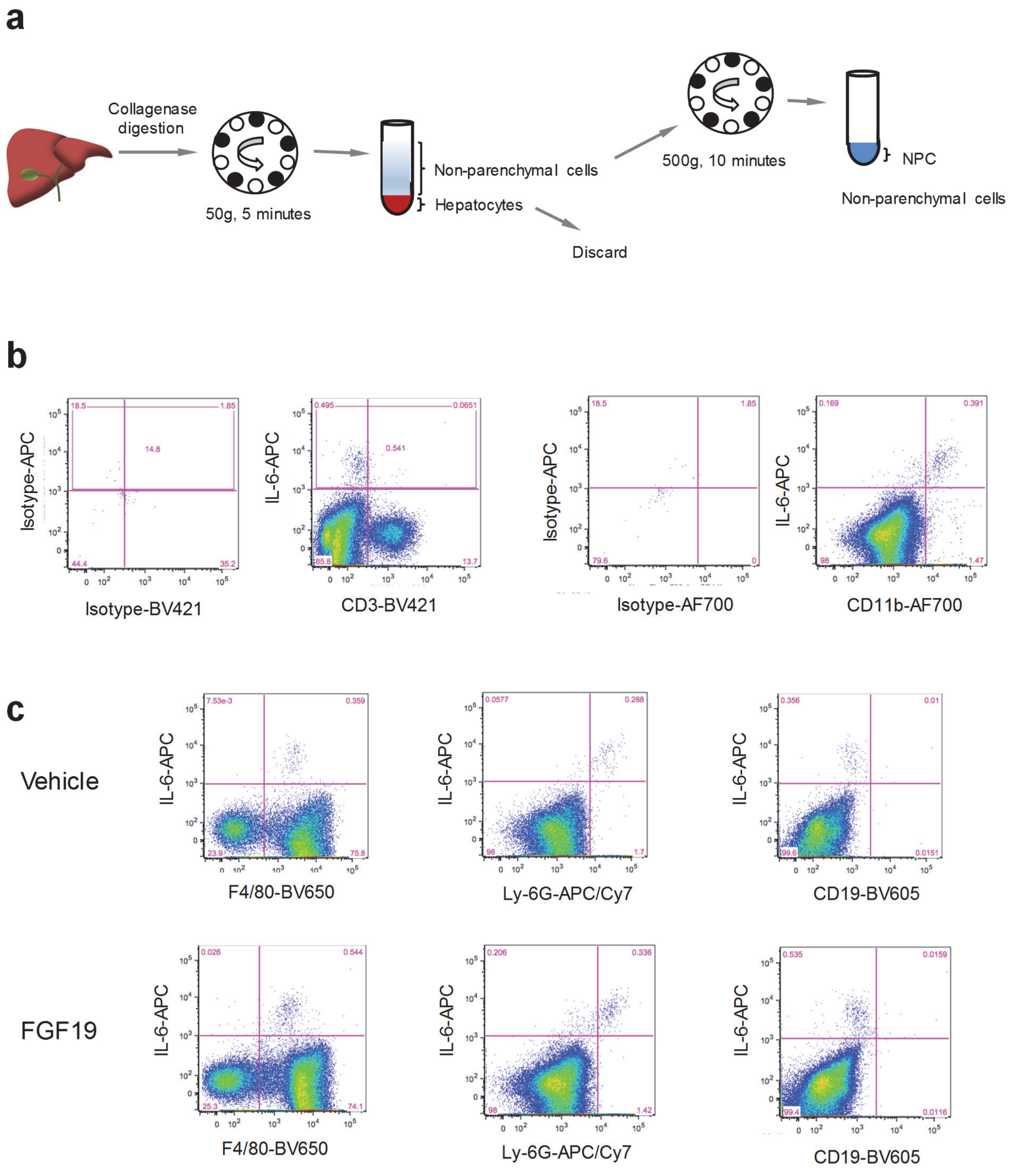
a
Collagenase digestion
50g, 5 minutes
Non-parenchymal cells
Hepatocytes
Discard
500g, 10 minutes
NPC
Non-parenchymal cells
b
Isotype-APC
Isotype-BV421
IL-6-APC
CD3-BV421
Isotype-APC
Isotype-AF700
IL-6-APC
CD11b-AF700
c
Vehicle
IL-6-APC
F4/80-BV650
IL-6-APC
Ly-6G-APC/Cy7
IL-6-APC
CD19-BV605
FGF19
IL-6-APC
F4/80-BV650
IL-6-APC
Ly-6G-APC/Cy7
IL-6-APC
CD19-BV605

d

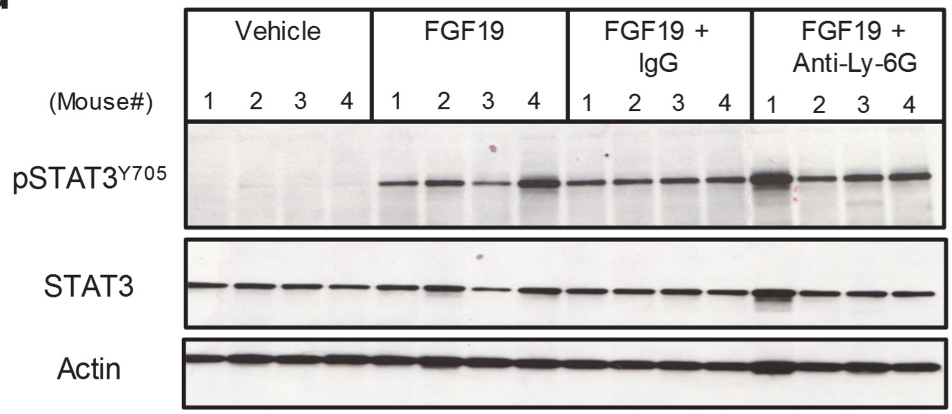
Vehicle
FGF19
FGF19 + IgG
FGF19 + Anti-Ly-6G
(Mouse#)
1 2 3 4 1 2 3 4 1 2 3 4 1 2 3 4
pSTAT3Y705
STAT3
Actin

e

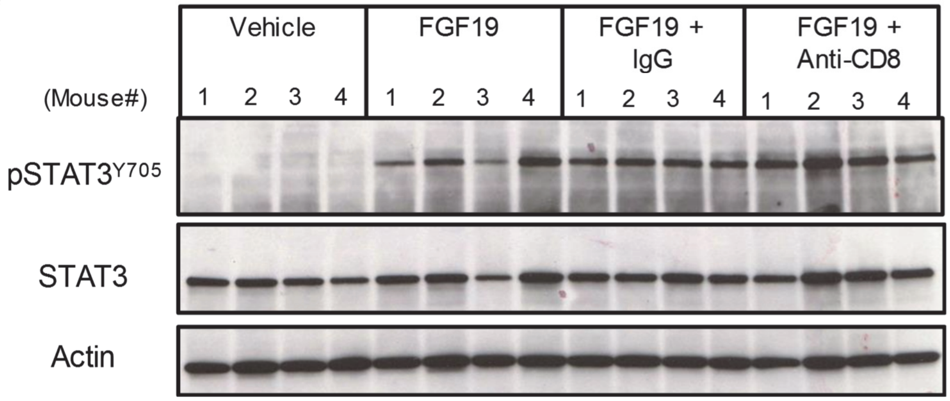
Vehicle
FGF19
FGF19 + IgG
FGF19 + Anti-CD8
(Mouse#)
1 2 3 4 1 2 3 4 1 2 3 4 1 2 3 4
pSTAT3Y705
STAT3
Actin

Supplementary Figure 5. Identification of secreted factor(s) mediating non-cell-autonomous activation of STAT3 by FGF19. (**a**) Schematic diagram of isolation of hepatic non-parenchymal (NPC) cells. (**b-c**) Intracellular IL-6 cytokine staining of non-parenchymal cells analyzed by flow cytometry. Isolated hepatic non-parenchymal cells were incubated in RPMI1640 media containing vehicle or 50 ng/mL FGF19 and protein transport inhibitors (10 μM brefeldin A and 2 mM monensin). Cells were subsequently stained with fluorophore-labeled antibodies (CD45, clone 30F-11; CD3, clone 17A2; CD19, clone 6D5; CD11b, clone M1/70; Ly6-G, clone 1A8; NK1.1, clone PK136; F4/80, clone BM8). Surface-stained cells were fixed and permeabilized, and incubated with anti-IL-6-APC (clone MP5-20F3). Staining with isotype control antibodies was included as negative controls in **b**. (**d-e**) Effect of immune cell depletion on FGF19-induced pSTAT3 activation. Antibody-mediated depletion of neutrophils (**d**) or CD8+ T cells (**e**) was performed prior to FGF19 injection in *db/db* mice (n = 4 per group). Isotype IgG serves as control. Livers were harvested 2 hours after FGF19 (1 mg/kg) injection. Shown are immunoblots of pSTAT3^{Y705}, total-STAT3 and anti-β-actin in liver lysates.

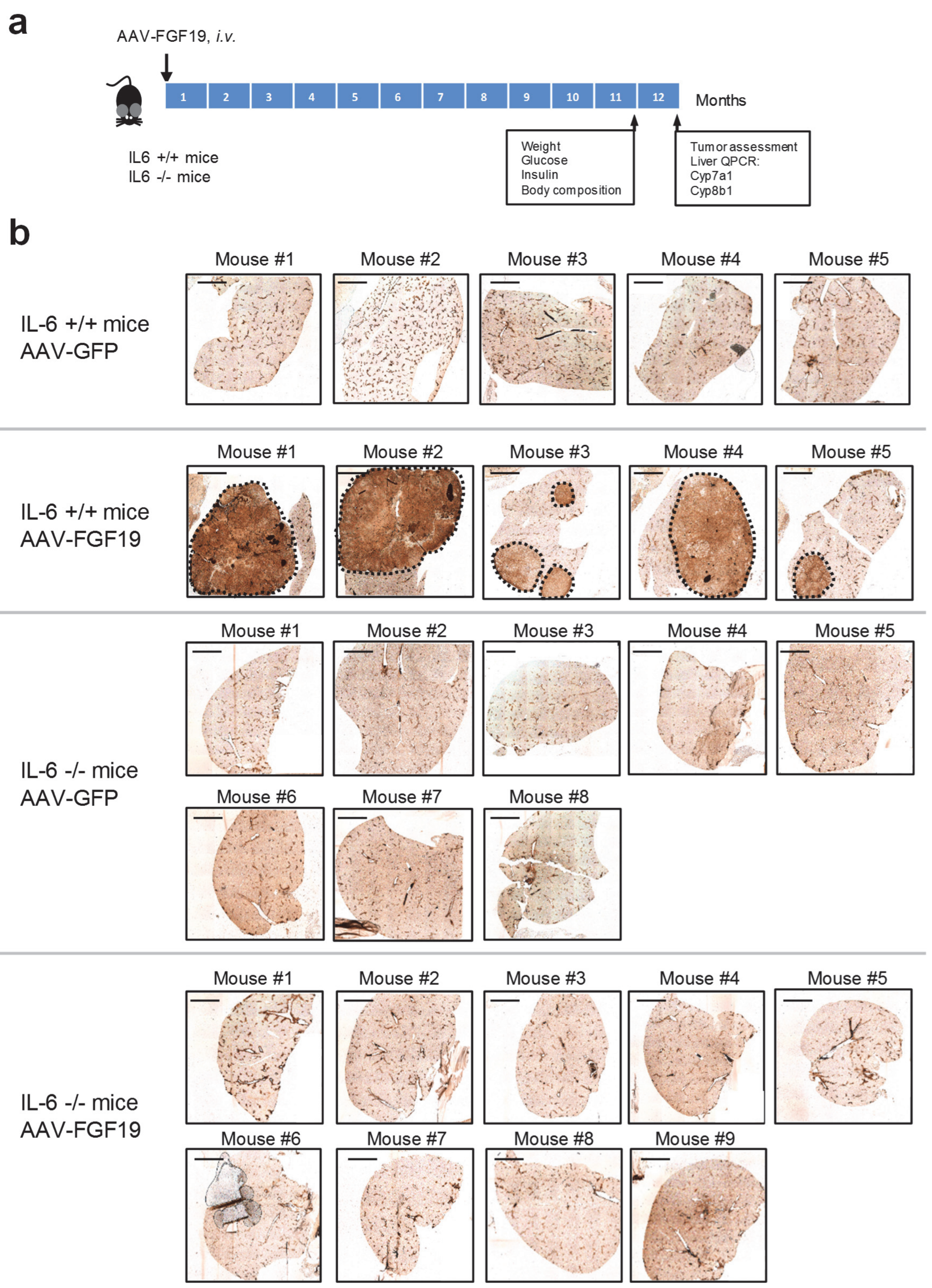
a
AAV-FGF19, i.v.
1
2
3
4
5
6
7
8
9
10
11
12
Months
IL6 +/+ mice
IL6 -/- mice
Weight
Glucose
Insulin
Body composition
Tumor assessment
Liver QPCR:
Cyp7a1
Cyp8b1
b
IL-6 +/+ mice
AAV-GFP
Mouse #1
Mouse #2
Mouse #3
Mouse #4
Mouse #5
IL-6 +/+ mice
AAV-FGF19
Mouse #1
Mouse #2
Mouse #3
Mouse #4
Mouse #5
IL-6 -/- mice
AAV-GFP
Mouse #1
Mouse #2
Mouse #3
Mouse #4
Mouse #5
Mouse #6
Mouse #7
Mouse #8
IL-6 -/- mice
AAV-FGF19
Mouse #1
Mouse #2
Mouse #3
Mouse #4
Mouse #5
Mouse #6
Mouse #7
Mouse #8
Mouse #9

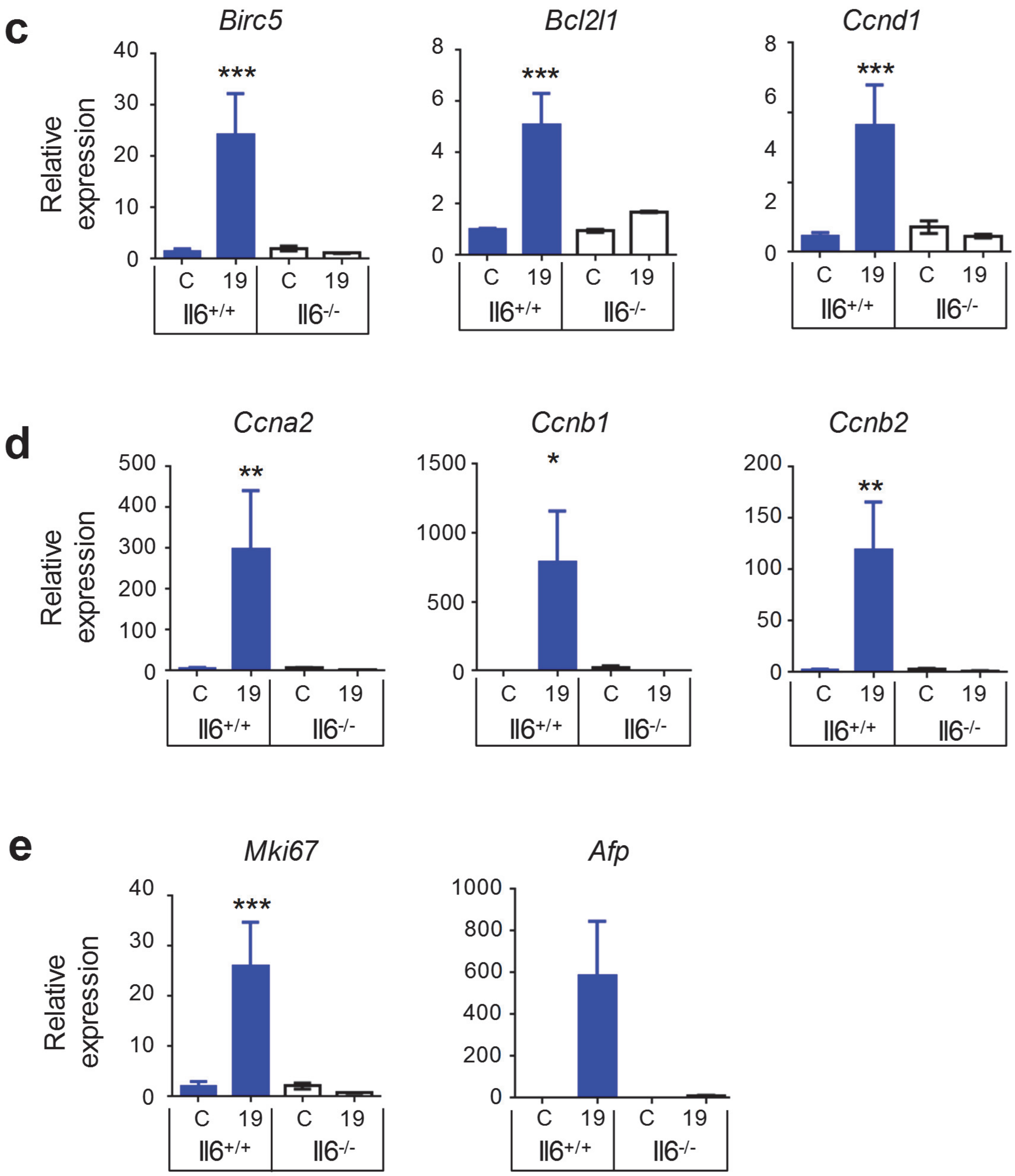
c
Birc5
Bcl2l1
Ccnd1
Relative expression
C 19 C 19
Il6+/+ Il6-/-
***
d
Ccna2
Ccnb1
Ccnb2
**
*
e
Mki67
Afp

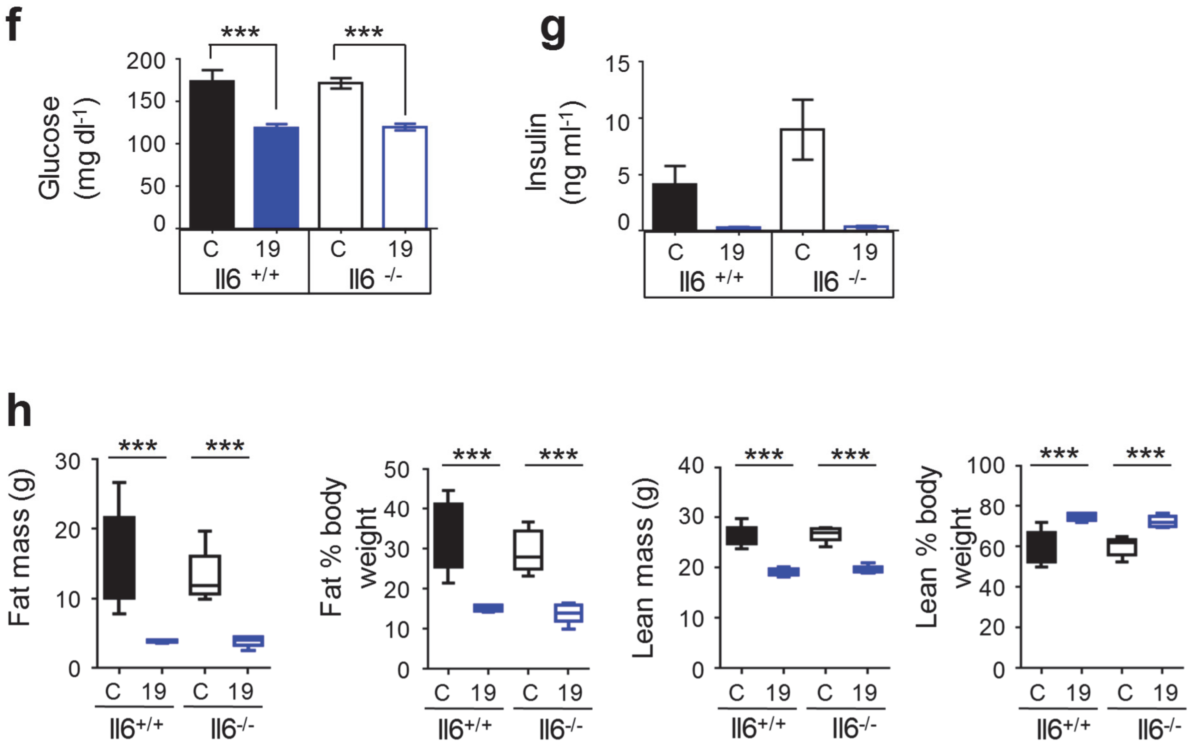

Supplementary Figure 6. Homozygous deletion of IL-6 prevents FGF19-induced HCC development while sparing FGF19-controlled metabolic improvements. (**a**) Study design. *Il6*$^{+/+}$ or *Il6*$^{-/-}$ mice received a single tail vein injection of AAV-FGF19 (19) or a control (C) virus. Body weight, glucose, insulin, and body composition were determined in live animals 11 months post-dose. Mice were sacrificed 12 months after AAV administration for tumor and gene expression analysis. Animal groups are *Il6*$^{+/+}$control (n = 5), *Il6*$^{+/+}$FGF19 (n = 5), *Il6*$^{-/-}$control (n = 8), *Il6*$^{-/-}$FGF19 (n = 9). (**b**) Tumor area for individual mouse by immunohistochemistry. Liver sections were stained with anti-glutamine synthetase and DAB substrates (brown color). Tumors are indicated by dashed lines; scale bars, 5 mm. (**c**) STAT3 target genes, *Birc5, Bcl2l1*, and *Ccnd1*, were induced by FGF19 in *Il6*$^{+/+}$ mice, but not *Il6*$^{-/-}$ mice. (**d**) Cyclins were induced by FGF19 in *Il6*$^{+/+}$ mice, but not *Il6*$^{-/-}$ mice. (**e**) FGF19 induces proliferative and HCC-related genes in *Il6*$^{+/+}$ mice, but not *Il6*$^{-/-}$ mice. (**f-h**) Glucose (**f**), insulin (**g**), and body composition (**h**) of the animals. Values are mean $\pm$ s.e.m. ***$P < 0.001$, **$P < 0.01$, *$P < 0.05$ versus control virus-injected *Il6*$^{+/+}$ mice by one-way ANOVA.

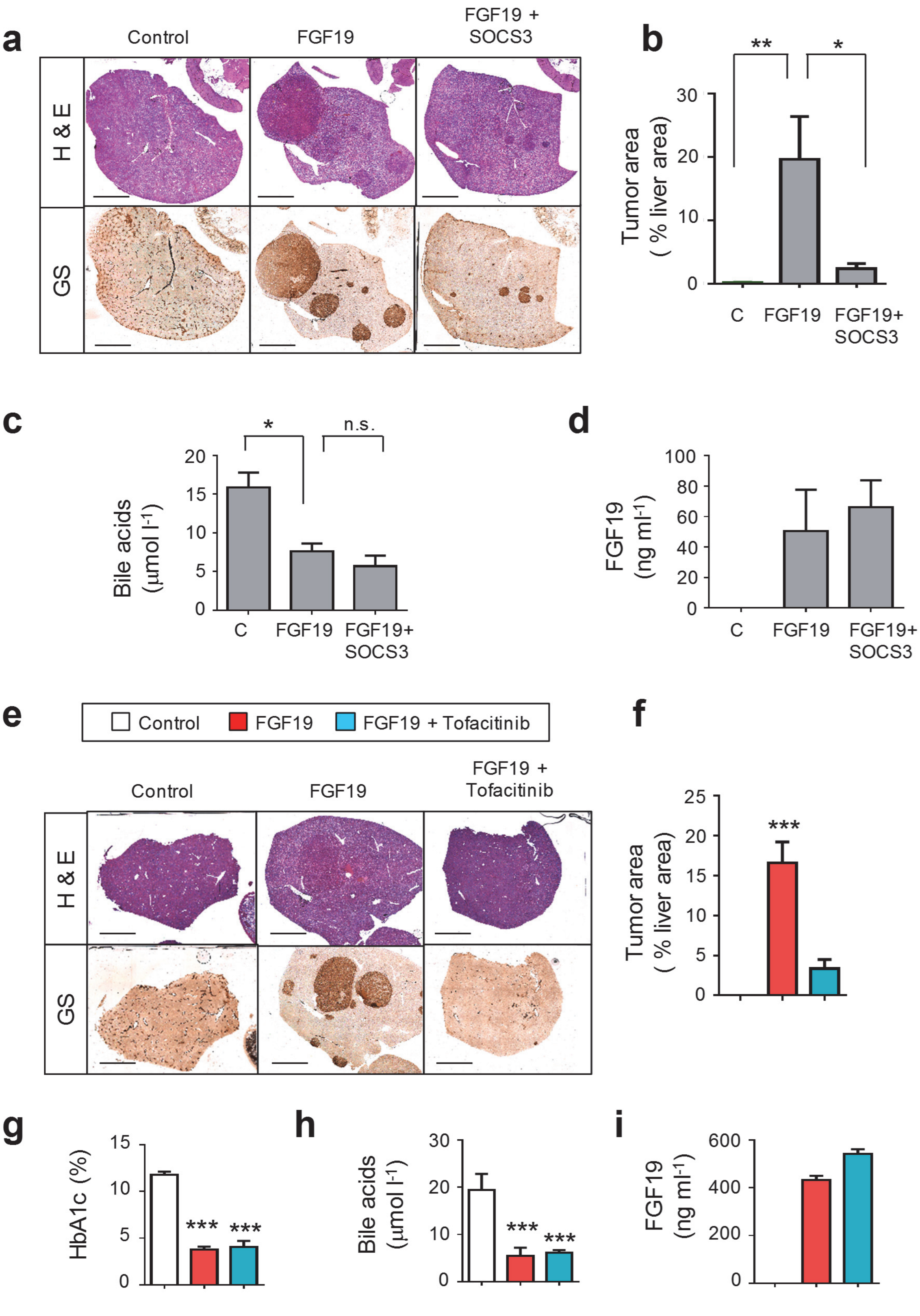
a
Control
FGF19
FGF19 + SOCS3
H & E
GS
b
**
*
Tumor area (% liver area)
30
20
10
0
C
FGF19
FGF19+ SOCS3
c
*
n.s.
Bile acids (µmol l-1)
20
15
10
5
0
C
FGF19
FGF19+ SOCS3
d
FGF19 (ng ml-1)
100
80
60
40
20
0
C
FGF19
FGF19+ SOCS3
e
Control
FGF19
FGF19 + Tofacitinib
Control
FGF19
FGF19 + Tofacitinib
H & E
GS
f
Tumor area (% liver area)
25
20
15
10
5
0
***
g
HbA1c (%)
15
10
5
0
***
***
h
Bile acids (µmol l-1)
30
20
10
0
***
***
i
FGF19 (ng ml-1)
600
400
200
0

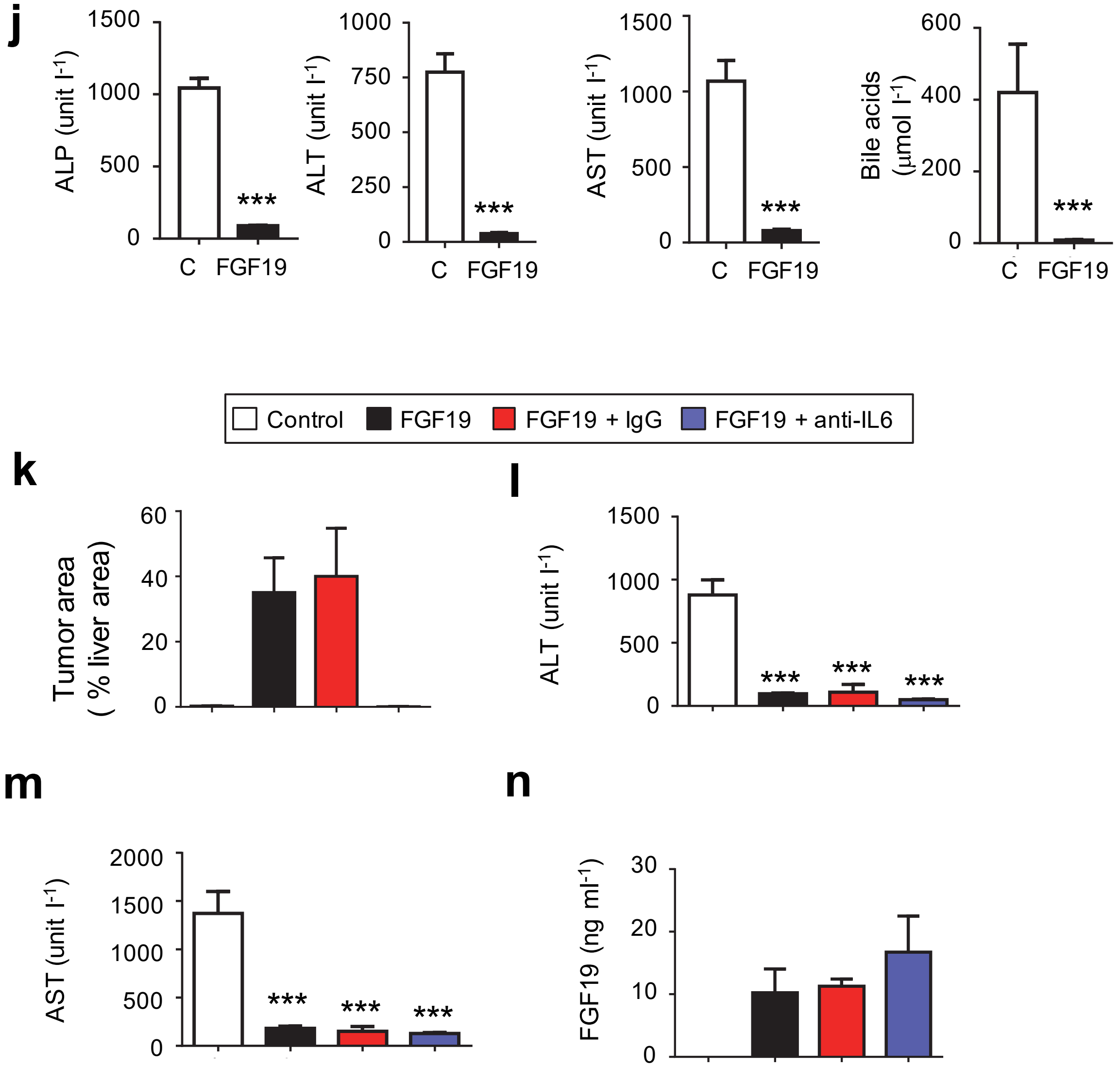
j
ALP (unit l-1)
ALT (unit l-1)
AST (unit l-1)
Bile acids (µmol l-1)
C
FGF19
***
Control
FGF19
FGF19 + IgG
FGF19 + anti-IL6
k
Tumor area (% liver area)
l
ALT (unit l-1)
m
AST (unit l-1)
n
FGF19 (ng ml-1)

Supplementary Figure 7. Pharmacological inhibition of the STAT3/IL-6 axis abolishes FGF19-dependent HCC. (a-d) AAV-SOCS3 study in *db/db* mice. 11~12-week old *db/db* mice (*n* = 5 per group) were *i.v.* administered with AAV-FGF19 with or without AAV-SOCS3, or a control virus. Mice were euthanized 24 weeks later for liver tumor analysis. Representative images of livers **(a)** (top panels, H&E staining; bottom panels, anti-glutamine synthetase staining with DAB substrates; scale bars, 5 mm), quantification of glutamine synthetase-positive tumor area **(b)**, serum levels of total bile acids **(c)** and FGF19 **(d)** are shown. **(e-i)** Tofacitinib study in *db/db* mice. 11~12-week old *db/db* mice (*n* = 5 per group) were *i.v.* administered with AAV-FGF19 or a control virus. Tofacitinib treatment was initiated 4 weeks later. Mice were euthanized 24 weeks after AAV injection. Representative images of livers **(e)** (top panels, H&E staining; bottom panels, anti-glutamine synthetase staining with DAB substrates; scale bars, 5 mm), quantification of glutamine synthetase-positive tumor area **(f)**, blood levels of HbA1c **(g)**, total bile acids **(h)** and FGF19 **(i)** are shown. **(j-n)** Anti-IL-6 study in $Mdr2^{-/-}$ mice. $Mdr2^{-/-}$ mice received a single tail vein injection of AAV-FGF19 or a control virus. Starting from week 14 after AAV injection, AAV-FGF19-injected mice were dosed intraperitoneally with 10 mg/kg anti-mouse IL-6 (*n* = 10) or an isotype control antibody (*n* = 8) weekly. Blood concentrations of liver enzymes were measured prior to antibody administration at week 14 **(j)**. At the end of the study, glutamine positive tumor areas were quantified **(k)**, and serum levels of ALT **(l)**, AST **(m)**, and FGF19 **(n)** were determined. Values are mean ± s.e.m. ***$P < 0.001$, **$P < 0.01$, *$P < 0.05$ by unpaired two-tailed t-test when comparing two groups, or versus control group by one-way ANOVA when comparing multiple groups.

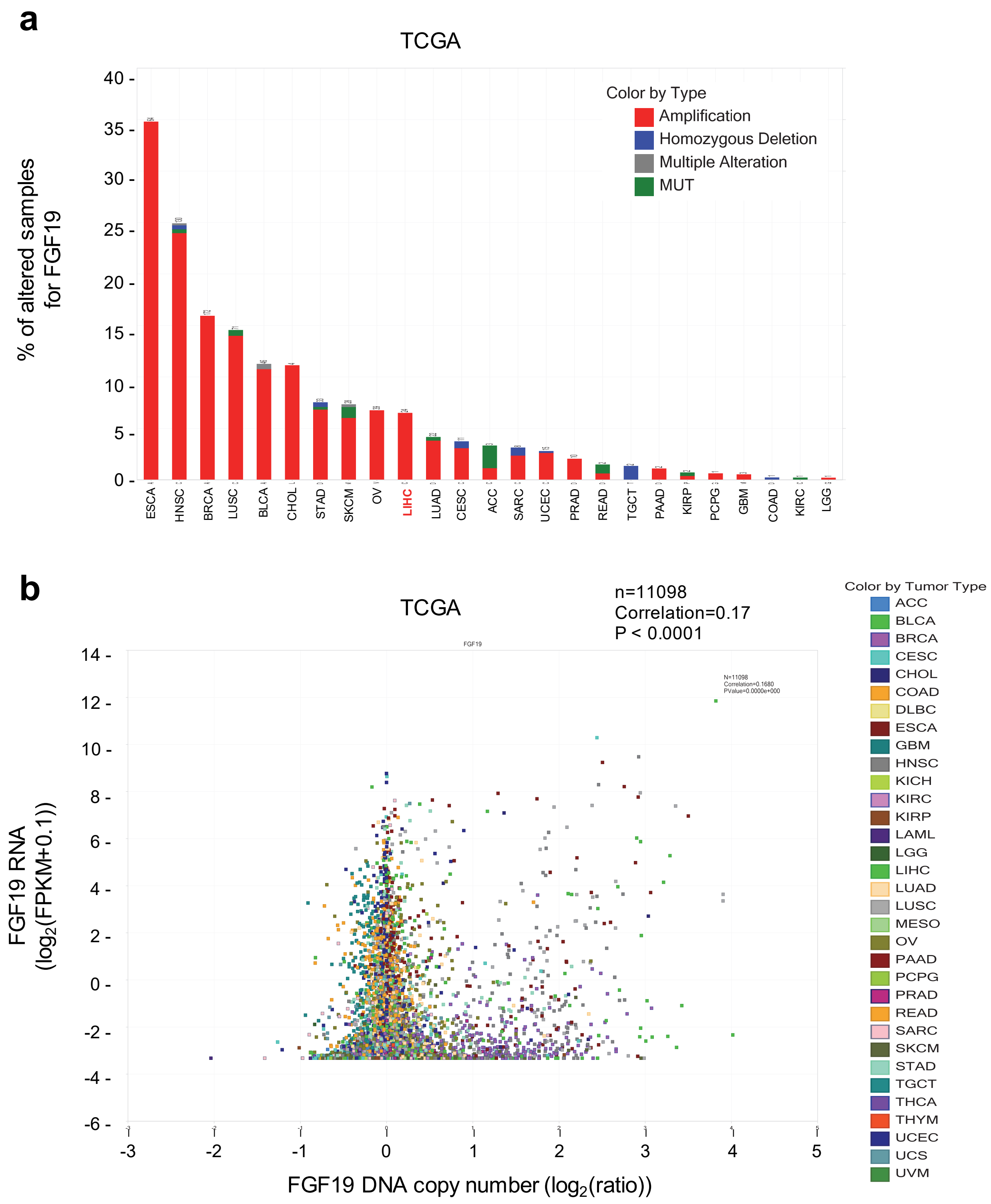

a
TCGA
% of altered samples for FGF19
Color by Type
Amplification
Homozygous Deletion
Multiple Alteration
MUT
ESCA
HNSC
BRCA
LUSC
BLCA
CHOL
STAD
SKCM
OV
LIHC
LUAD
CESC
ACC
SARC
UCEC
PRAD
READ
TGCT
PAAD
KIRP
PCPG
GBM
COAD
KIRC
LGG
b
TCGA
n=11098
Correlation=0.17
P < 0.0001
FGF19 RNA (log2(FPKM+0.1))
FGF19 DNA copy number (log2(ratio))
Color by Tumor Type
ACC
BLCA
BRCA
CESC
CHOL
COAD
DLBC
ESCA
GBM
HNSC
KICH
KIRC
KIRP
LAML
LGG
LIHC
LUAD
LUSC
MESO
OV
PAAD
PCPG
PRAD
READ
SARC
SKCM
STAD
TGCT
THCA
THYM
UCEC
UCS
UVM

c

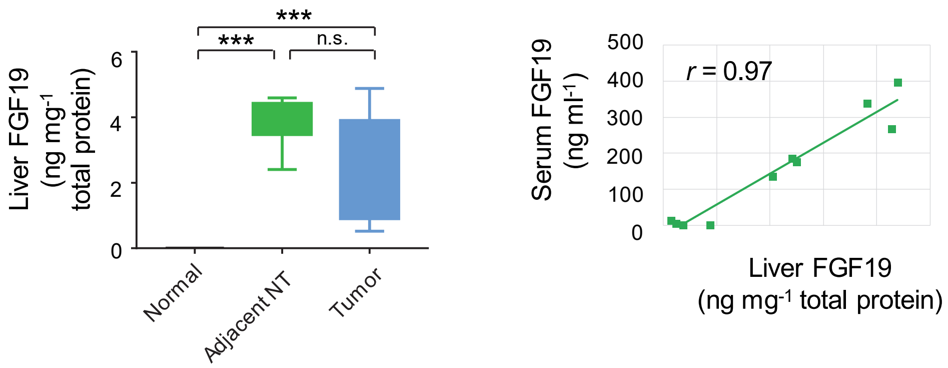
***
***
n.s.
Liver FGF19
(ng mg-1
total protein)
0
2
4
6
Normal
Adjacent NT
Tumor
Serum FGF19
(ng ml-1)
0
100
200
300
400
500
r = 0.97
Liver FGF19
(ng mg-1 total protein)

d

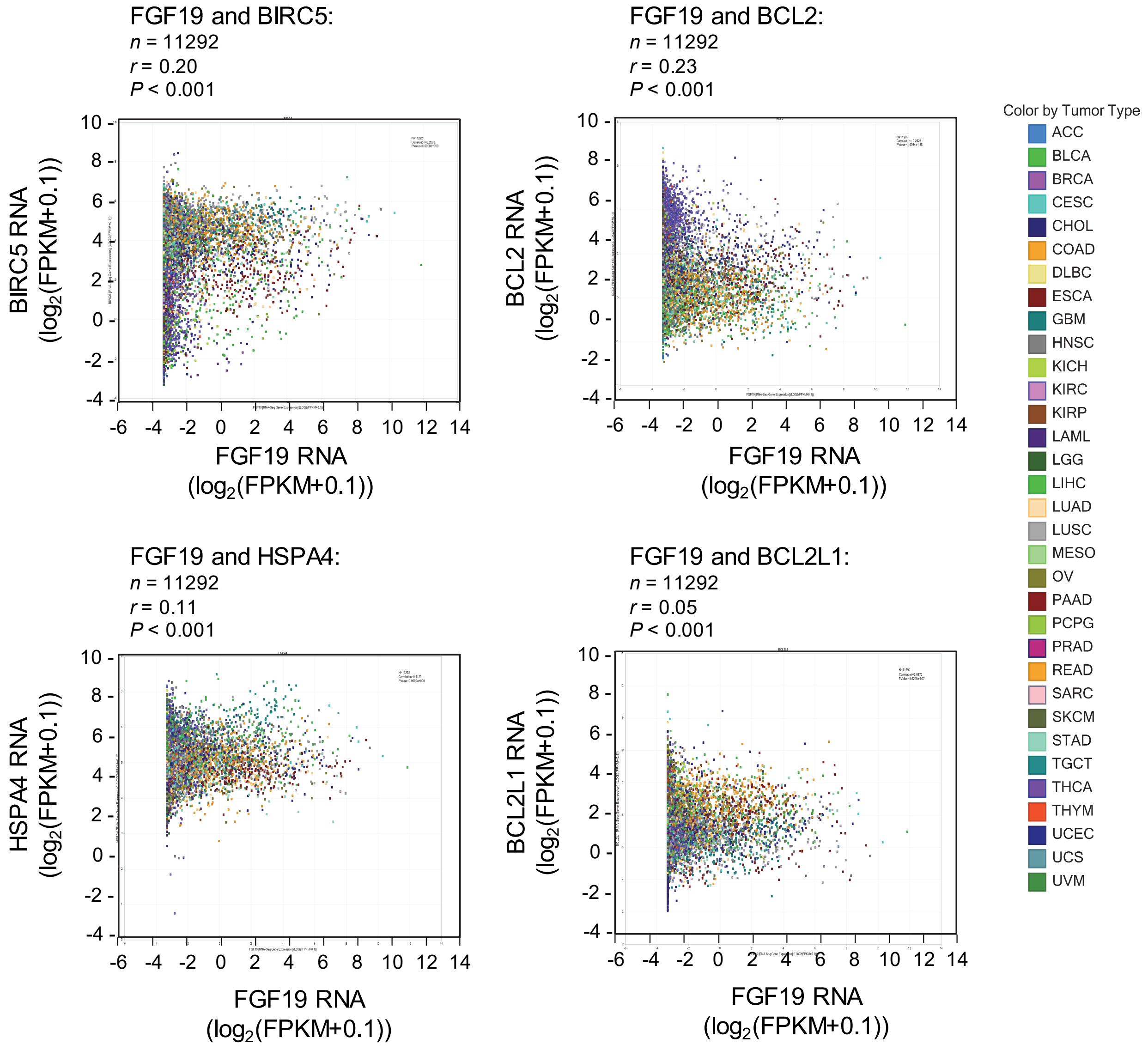
FGF19 and BIRC5:
n = 11292
r = 0.20
P < 0.001
BIRC5 RNA
(log2(FPKM+0.1))
FGF19 RNA
(log2(FPKM+0.1))
FGF19 and BCL2:
n = 11292
r = 0.23
P < 0.001
BCL2 RNA
(log2(FPKM+0.1))
FGF19 RNA
(log2(FPKM+0.1))
FGF19 and HSPA4:
n = 11292
r = 0.11
P < 0.001
HSPA4 RNA
(log2(FPKM+0.1))
FGF19 RNA
(log2(FPKM+0.1))
FGF19 and BCL2L1:
n = 11292
r = 0.05
P < 0.001
BCL2L1 RNA
(log2(FPKM+0.1))
FGF19 RNA
(log2(FPKM+0.1))
-6 -4 -2 0 2 4 6 8 10 12 14
-4 -2 0 2 4 6 8 10
Color by Tumor Type
ACC
BLCA
BRCA
CESC
CHOL
COAD
DLBC
ESCA
GBM
HNSC
KICH
KIRC
KIRP
LAML
LGG
LIHC
LUAD
LUSC
MESO
OV
PAAD
PCPG
PRAD
READ
SARC
SKCM
STAD
TGCT
THCA
THYM
UCEC
UCS
UVM

Supplementary Figure 8. Genetic alterations and correlations in FGF19 in human cancers. **(a)** Frequency of FGF19 genetic alterations in human tumors from the Cancer Genome Atlas (TCGA) database. Types of alterations include amplification, homozygous deletion, mutation and multiple alterations. Genetic alterations in genes encoding CTNNB1, PTEN and TP53 are shown for comparison. **(b)** Scatter plot of FGF19 DNA copy number versus mRNA expression in TCGA database. Spearman correlation coefficient (*r*) and *P* value are displayed. mRNA levels retrieved from RNA-seq data are expressed as Fragments Per Kilobase of transcript per Million mapped reads (FPKM). **(c)** Quantification of FGF19 levels in liver tumors and adjacent non-tumor (NT) liver tissues in mice injected with AAV-FGF19. Note that FGF19 is undetectable in normal livers from untreated mice. FGF19 concentrations in liver extracts were determined by ELISA, and expressed as ng per mg of total liver protein. Scatter plot shows serum FGF19 concentrations correlate with FGF19 levels in the liver in mice injected with AAV-FGF19. ****P* < 0.001; n.s., not statistically significant. **(d)** FGF19 mRNA levels correlate with the expression of STAT3 target genes in human cancers in TCGA database. Correlation of RNA FPKM for FGF19 and BIRC5, BCL2, HSPA4, or BCL2L1 is shown as scatter plots, with each tumor type indicated on the right. Spearman correlation coefficient (*r*) and *P* value are displayed.

Figure 3b

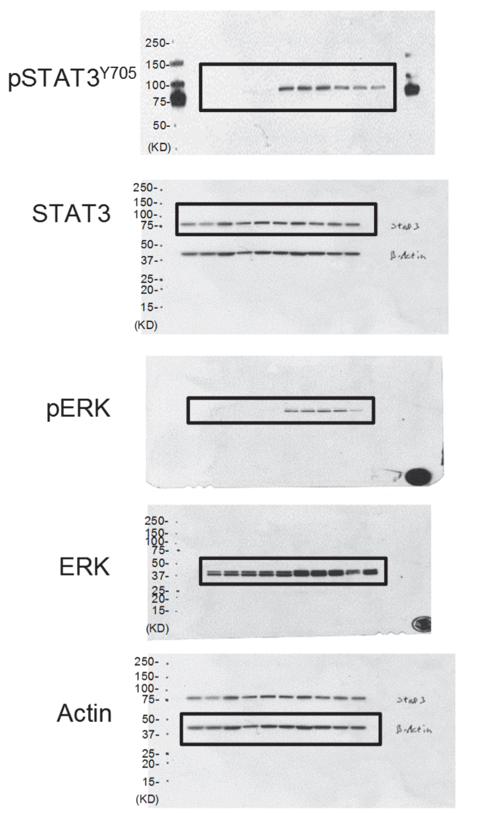

Figure 3d

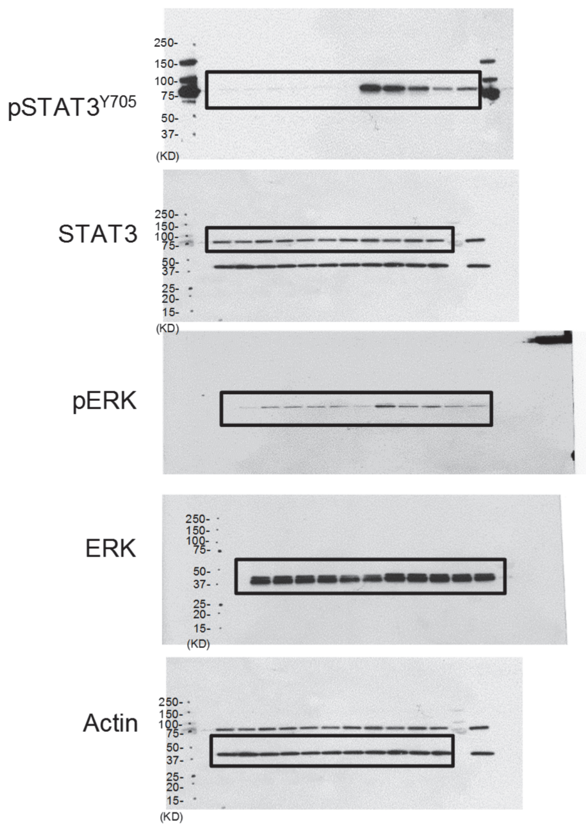

Figure 3e

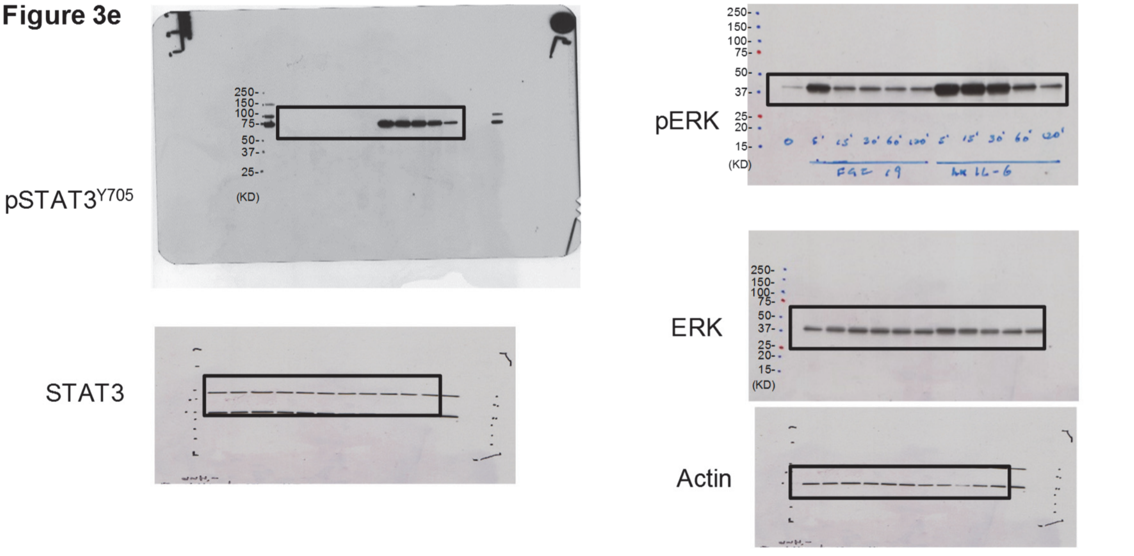

Figure 5e

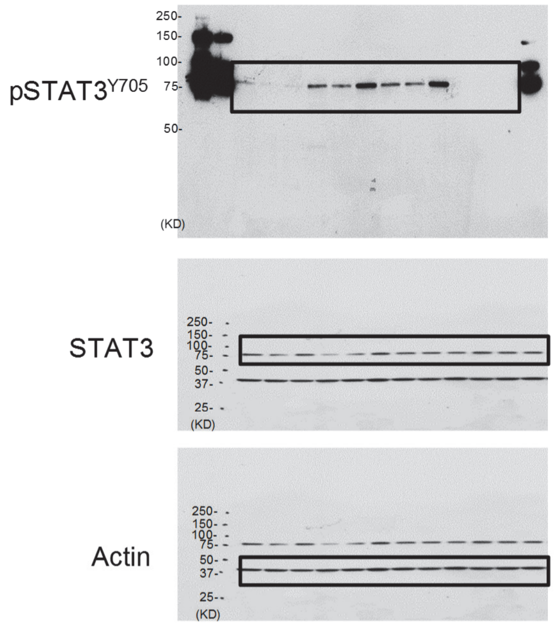

Figure 5h

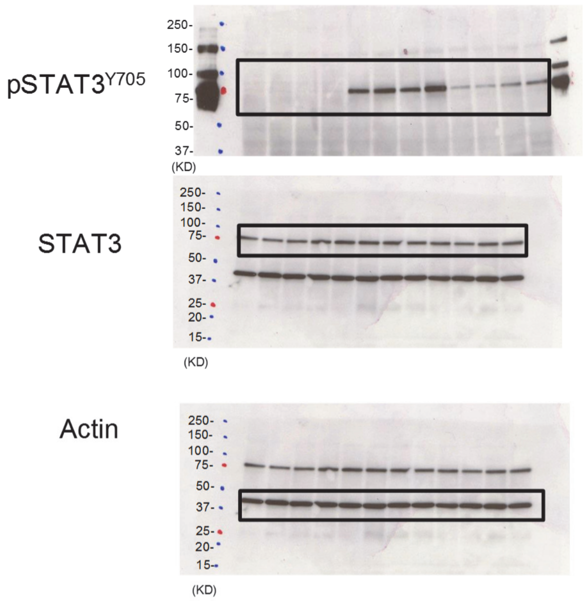

Supplementary Figure 3a

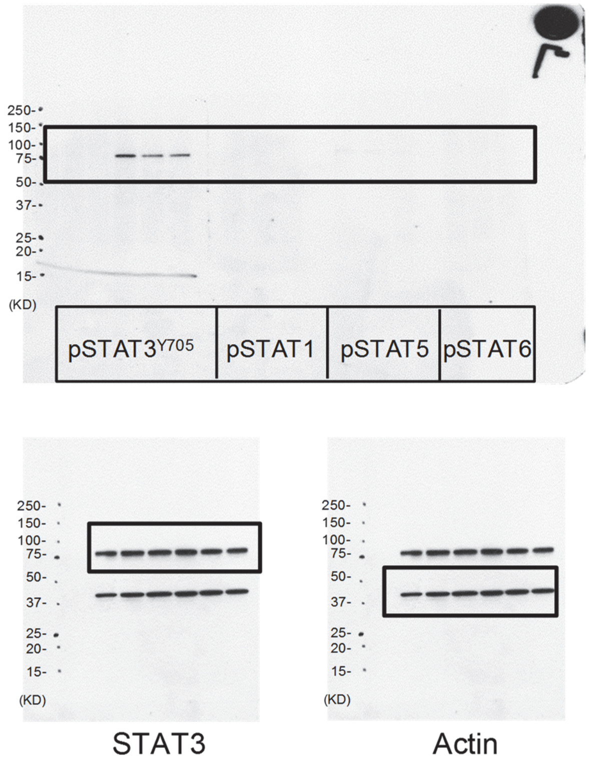

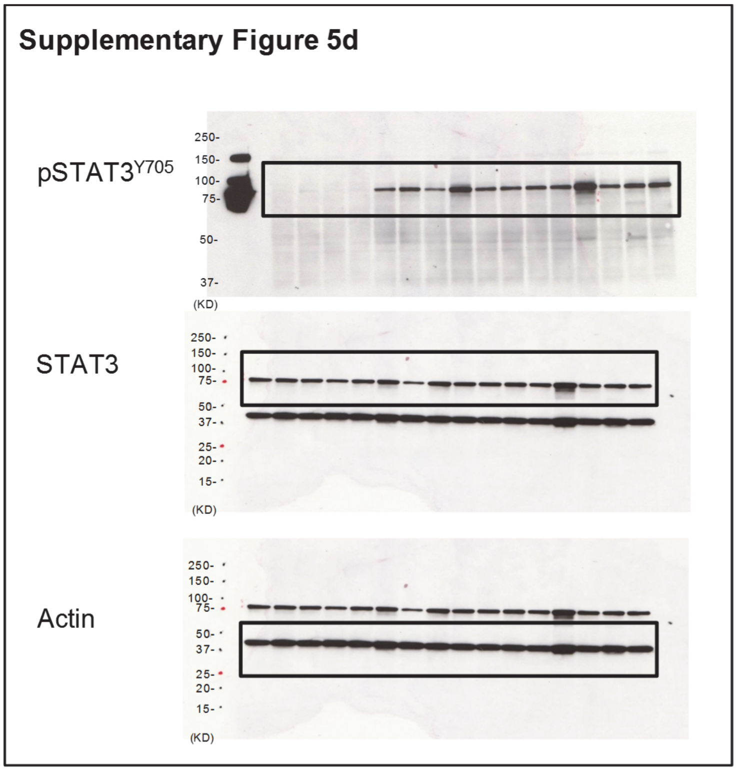

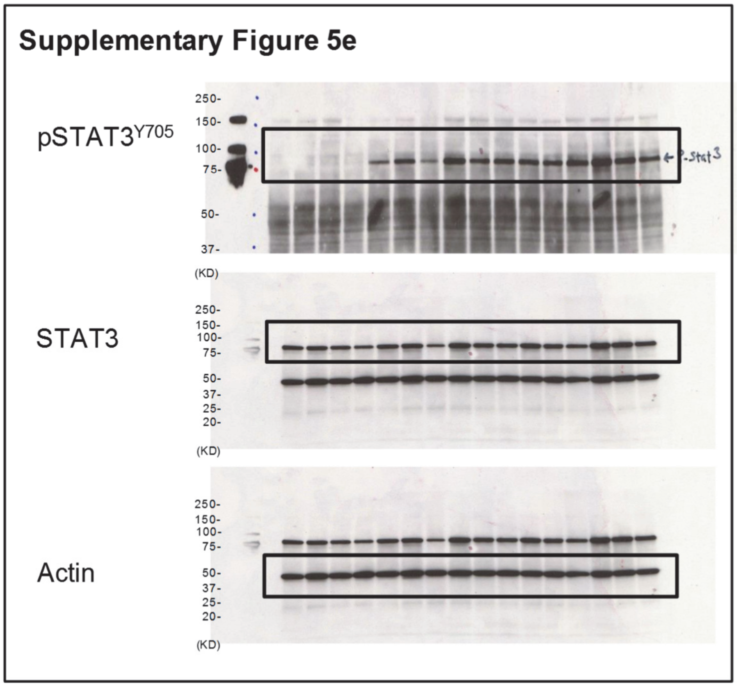

Supplementary Figure 9. Uncropped immunoblots with markers of molecular weights.

Supplementary Table 1. TCGA datasets used in the current study

Tumor Type Abbreviations	Tumor Type Full Name	Number of Patient Samples
ACC	Adrenocortical carcinoma	184
BLCA	Bladder Urothelial Carcinoma	846
BRCA	Breast invasive carcinoma	2298
CESC	Cervical squamous cell carcinoma and endocervical adenocarcinoma	620
CHOL	Cholangiocarcinoma	115
COAD	Colon adenocarcinoma	989
DLBC	Lymphoid Neoplasm Diffuse Large B-cell Lymphoma	116
ESCA	Esophageal carcinoma	377
GBM	Glioblastoma multiforme	1174
HNSC	Head and Neck squamous cell carcinoma	1123
KICH	Kidney Chromophobe	226
KIRC	Kidney renal clear cell carcinoma	1101
KIRP	Kidney renal papillary cell carcinoma	614
LAML	Acute Myeloid Leukemia	699
LGG	Brain Lower Grade Glioma	1051
LIHC	Liver hepatocellular carcinoma	796
LUAD	Lung adenocarcinoma	1288
LUSC	Lung squamous cell carcinoma	1083
MESO	Mesothelioma	174
OV	Ovarian serous cystadenocarcinoma	1221
PAAD	Pancreatic adenocarcinoma	377
PCPG	Pheochromocytoma and Paraganglioma	366
PRAD	Prostate adenocarcinoma	1063
READ	Rectum adenocarcinoma	349
SARC	Sarcoma	531
SKCM	Skin Cutaneous Melanoma	951
STAD	Stomach adenocarcinoma	940
TGCT	Testicular Germ Cell Tumors	306
THCA	Thyroid carcinoma	1053
THYM	Thymoma	250
UCEC	Uterine Corpus Endometrial Carcinoma	1131
UCS	Uterine Carcinosarcoma	114
UVM	Uveal Melanoma	160

Supplementary Table 2. Patient information for duplex (FGF19 + BIRC5) RNAScope *in situ hybridization* on formalin-fixed, paraffin-embedded normal liver tissues

Subject ID	Gender	Age	Organ	Pathology	Grade	Stage	TNM	Type
1	F	14	Liver	Normal hepatic tissue	-	-	-	Normal
2	F	50	Liver	Normal hepatic tissue	-	-	-	Normal
3	F	35	Liver	Normal hepatic tissue	-	-	-	Normal
4	M	40	Liver	Normal hepatic tissue	-	-	-	Normal
5	M	40	Liver	Normal hepatic tissue	-	-	-	Normal
6	M	38	Liver	Normal hepatic tissue	-	-	-	Normal
7	M	45	Liver	Normal hepatic tissue	-	-	-	Normal
8	F	18	Liver	Normal hepatic tissue	-	-	-	Normal
9	M	43	Liver	Normal hepatic tissue	-	-	-	Normal
10	M	16	Liver	Normal hepatic tissue	-	-	-	Normal

Supplementary Table 3. Patient information for quantitative RT-PCR analysis of STAT3 target genes on RNA extracted from frozen normal liver tissues, FGF19–expressing, and FGF19-non-expressing HCCs

Subject ID	Gender	Age	Organ	Pathology	Source	Catalog Number	Type	FGF19
N1	M	53	Liver	Normal hepatic tissue	Bioreclamation	MHU-L-112210	Normal	
N2	M	49	Liver	Normal hepatic tissue	Bioreclamation	MHU-L-040211	Normal	
N3	M	38	Liver	Normal hepatic tissue	Bioreclamation	MHU-L-071907	Normal	
N4	M	37	Liver	Normal hepatic tissue	Bioreclamation	MHU-L-122108	Normal	
N5	M	62	Liver	Normal hepatic tissue	Bioreclamation	MHU-L-100510	Normal	
T1	F	51	Liver	Hepatocellular carcinoma	CrownBio	LI0574	Malignant	FGF19-expressing
T2	M	59	Liver	Hepatocellular carcinoma	CrownBio	LI1054	Malignant	FGF19-expressing
T3	M	53	Liver	Hepatocellular carcinoma	CrownBio	LI1081	Malignant	FGF19-expressing
T4	M	65	Liver	Hepatocellular carcinoma	CrownBio	LI6619	Malignant	FGF19-expressing
T5	M	45	Liver	Hepatocellular carcinoma	CrownBio	LI6677	Malignant	FGF19-expressing
T6	M	NA	Liver	Hepatocellular carcinoma	CrownBio	LI0801	Malignant	FGF19-non-expressing
T7	M	53	Liver	Hepatocellular carcinoma	CrownBio	LI1025	Malignant	FGF19-non-expressing
T8	F	56	Liver	Hepatocellular carcinoma	CrownBio	LI1037	Malignant	FGF19-non-expressing
T9	M	33	Liver	Hepatocellular carcinoma	CrownBio	LI1097	Malignant	FGF19-non-expressing
T10	M	48	Liver	Hepatocellular carcinoma	CrownBio	LI6686	Malignant	FGF19-non-expressing

Supplementary Table 4. List of antibodies used for immunohistochemistry and immunoblotting

Antigen	Vendor	Catalog Number	Host Species	Dilution
For immunohistochemistry:				
BrdU	Dako	M0744	Mouse IgG	1:500
Glutamine synthetase	Abcam	Ab49873	Rabbit IgG	1:2000
Ki-67	Dako	M7249	Mouse IgG	1:500
PCNA	Dako	M0879	Mouse IgG	1:200
STAT3 (total)	Cell Signaling Technology	4904	Rabbit IgG	1:1000
Biotinylated anti-mouse IgG	Vector Laboratories	BA-2000	Horse IgG	1:500
Biotinylated anti-rabbit IgG	Vector Laboratories	BA-1000	Goat IgG	1:500
For immunoblotting:				
β-Actin	Sigma	A5441	Mouse IgG	1:10000
ERK1/2 (total)	Santa Cruz	sc-94	Rabbit IgG	1:200
$pERK1/2^{T202Y204}$	Cell Signaling Technology	4370	Rabbit IgG	1:2000
$pSTAT1^{Y701}$	Cell Signaling Technology	7649	Rabbit IgG	1:1000
$pSTAT3^{Y705}$	Cell Signaling Technology	9145	Rabbit IgG	1:1000
$pSTAT5^{Y694}$	Cell Signaling Technology	4322	Rabbit IgG	1:1000
$pSTAT6^{Y641}$	Cell Signaling Technology	9361	Rabbit IgG	1:1000
STAT3 (total)	Cell Signaling Technology	4904	Rabbit IgG	1:2000

Supplementary Table 5. List of antibodies used for flow cytometry

Antigen	Clone	Vendor	Catalog Number	Dilution
CD3-BV421	17A2	BioLegend	100228	1:40
Rat IgG2b-BV421		BioLegend	400655	1:40
CD11b-AF700	M1/70	eBioscience	56-0112	1:40
Rat IgG2b-AF700		BioLegend	400628	1:40
CD19-BV605	6D5	BioLegend	115540	1:40
Rat IgG2a-BV605		BioLegend	400540	1:40
CD45-FITC	30-F11	BioLegend	103108	1:100
Rat IgG2b-FITC		BioLegend	400606	1:100
F4/80-BV650	BM8	BioLegend	123149	1:40
Rat IgG2a-BV650		BioLegend	400542	1:40
IL-6-APC	MP5-20F3	BioLegend	504507	1:40
Rat IgG1-APC		BioLegend	400411	1:40
Ly-6G-APC/Cy7	1A8	BioLegend	127624	1:40
Rat IgG2a-APC/Cy7		BioLegend	400524	1:40
NK1.1-PE/Cy7	PK136	BioLegend	108714	1:40
